# AI and Ethics When Human Beings Collaborate With AI Agents

**DOI:** 10.3389/fpsyg.2022.836650

**Published:** 2022-03-04

**Authors:** José J. Cañas

**Affiliations:** Mind, Brain and Behaviour Research Centre, University of Granada, Granada, Spain

**Keywords:** AI, ethics, agent collaboration, human-AI interaction, human factors

## Abstract

The relationship between a human being and an AI system has to be considered as a collaborative process between two agents during the performance of an activity. When there is a collaboration between two people, a fundamental characteristic of that collaboration is that there is co-supervision, with each agent supervising the actions of the other. Such supervision ensures that the activity achieves its objectives, but it also means that responsibility for the consequences of the activity is shared. If there is no co-supervision, neither collaborator can be held co-responsible for the actions of the other. When the collaboration is between a person and an AI system, co-supervision is also necessary to ensure that the objectives of the activity are achieved, but this also means that there is co-responsibility for the consequences of the activities. Therefore, if each agent's responsibility for the consequences of the activity depends on the effectiveness and efficiency of the supervision that that agent performs over the other agent's actions, it will be necessary to take into account the way in which that supervision is carried out and the factors on which it depends. In the case of the human supervision of the actions of an AI system, there is a wealth of psychological research that can help us to establish cognitive and non-cognitive boundaries and their relationship to the responsibility of humans collaborating with AI systems. There is also psychological research on how an external observer supervises and evaluates human actions. This research can be used to programme AI systems in such a way that the boundaries of responsibility for AI systems can be established. In this article, we will describe some examples of how such research on the task of supervising the actions of another agent can be used to establish lines of shared responsibility between a human being and an AI system. The article will conclude by proposing that we should develop a methodology for assessing responsibility based on the results of the collaboration between a human being and an AI agent during the performance of one common activity.

## Introduction

Most approaches to the ethics of AI have been made to answer questions about the ethical principles that should guide the development of AI systems. Thus, much thought and research has been given to what can be designed, to the purpose for which it should be designed, to the limits that should be placed on the design of AI so that these systems do not cause harm to humans, and so on (Stahl et al., 2017). We might call this approach the “ethics of design goals.” However, we also need to address another very important aspect of ethics and AI: the responsibilities of the people and the AI systems with which they interact, and the consequences of their joint actions. We have to recognize that, by using the term “intelligence,” we are assuming that AI systems are agents that “collaborate” with other human and artificial agents in performing a task. Therefore, the ethical implications of the results of this collaborative task have to be considered from the point of view of the collaboration between the agents, and the agents' common responsibilities. Following this approach, what we have to do is to ask ourselves what the ethical responsibilities are for a good or bad result of individual actions in the context of a system in which AI and human agents jointly collaborate. This approach would be along the lines of what has been called “collective responsibility” (French and Wettstein, 2006).

This is the philosophical approach taken by authors such as Floridi (2016). This author has explored the ethical issues related to the outcomes of joint actions taken by several interacting agents in the performance of a task. He speaks of “distributed moral responsibility,” which is the consequence of “distributed moral actions.” On these issues, Floridi identifies two main points of view that can be taken to address distributed moral responsibility. One view is what he calls “agent-oriented ethics,” in which the emphasis is on the actions of each individual agent and the interest is in the individual development, social welfare, and ultimate salvation of each agent. From this point of view, one should speak of the individual responsibility of each agent for the results of their individual actions.

Another point of view is what this author calls “patient-oriented ethics,” which is concerned with the ultimate welfare of the system. The final results of the individual actions of individual agents within a system in which they collaborate have consequences for the environment in which the system is located. The word “patient” that Floridi uses refers to the entity who receives the effects of the overall actions of the system. We can think of a system such as a hospital in which many agents (healthcare workers with different specialities) collaborate and in which there is one external agent, the patient, who receives the effects of the collaborative actions of all the healthcare workers. To give another example related to AI, we can imagine that we have a car that is driven jointly by an intelligent system installed in it and by a human driver. When there is an accident, the agent-oriented approach to ethics would be concerned with analyzing the ethical responsibilities of the individual actors, who in this case are the artificial intelligence system in the car and the human driver. However, patient-based ethics would be concerned with the ethical responsibilities of the system consisting of the car and the human driver as a whole, with respect to the consequences of the system's actions on the surrounding environment (e.g., pedestrians).

What is important is that an agent-oriented ethics aims to analyse the morality of the individual actions of agents to whom intentionality is attributed. In the example of the car, when analyzing an accident we analyse the morality of the actions of the individual agents (the AI system and the human driver) individually. We assume that the accident (the morally negative event) could have come from the morally negative actions of one of the agents, or of both of the agents separately. Thus, the engineers designing the car's AI system will try to ensure that the actions of the AI system follow ethical criteria, but without taking into account the possible actions of the other agent, the human driver. In the same way, driving teachers will train drivers to drive according to the ethical rules by which a human being must be governed, but without considering how the AI agent is designed to act.

However, in patient-oriented ethics (e.g., when looking at road accidents with a focus on pedestrians) we can do a “backwards” analysis in which, when the system (the car with the human driver and the AI system) performs a morally negative action (e.g., hitting a pedestrian), we assume that the individual actions were not morally negative when considered by themselves. The consequences of the morally negative action of the system were the result of a wrong interaction of the two agents that collaborate in the task of driving. For example, the human driver could misinterpret the action of the intelligent system and act incorrectly because of this misinterpretation. Therefore, the responsibility does not lie with one of the individual agents (whom we can assume had good intentionality) but with the bad design of the interaction.

Before going any further, it is necessary to define three concepts that tend to be confused because, although in English the words for these concepts have different meanings, the concepts are all related. The confusion may be due to the fact that in other languages such as Spanish or French there is only one word used to refer to all three concepts together. The three concepts are accountability, responsibility, and liability. The (Encyclopaedia Britannica, 2021) defines accountability as the “principle according to which a person or institution is responsible for a set of duties and can be required to give an account of their fulfillment to an authority that is in a position to issue rewards or punishment.” Thus, accountability fundamentally means being able to give an explanation for one's behavior. Therefore, when a person and an AI agent collaborate in an activity, each of them is accountable if they are able to give an explanation of their individual actions, but we can also speak of the accountability of the joint system formed by the two agents, the human being and the AI agent, to the extent that they are able to explain their joint behavior.

A concept related to accountability is responsibility which, according to the (Encyclopaedia Britannica, 2021), is “the technical term that was preferred to indicate the duty that persons in public authority had to ‘respond’ in their conduct and actions as public officials.” A term related to responsibility in the legal environment is liability, which the Encyclopaedia Britannica defines as the concept “preferred to indicate that by doing a certain action (or entering into a certain contract) a person has put himself under an obligation and is therefore answerable for the consequences following from that action (or from entering into that contract).”

This clarification is important because in other languages there is only one word for all three concepts. That word in Spanish, for example, is *responsabilidad*. This often causes speakers of languages other than English to use the English word “responsibility” without clarifying whether they mean accountability, responsibility, or liability, creating confusion in the discussion of ethics and AI. What we are interested in here is accountability and, to a lesser extent, responsibility, but we are not interested in liability, which is a concept that is of interest only in the legal sphere. If we are talking about an accident involving a car with a human driver and an AI system, we are not interested in the legal consequences of that accident. What we are interested in here is the extent to which the two agents, the human driver and the AI system installed in the car, can explain their behavior and be held “jointly” responsible for it.

Our interest in this article is therefore in the ethics of the collaboration between an AI system and a human agent. This is the more common point of view of human factors and ergonomics. For human factors specialists, the interest is in the design of the interaction between an AI agent that interacts with a human being in a joint activity (such as driving). That interaction should be designed while thinking that what is important is the design of the interaction and not the design of each agent separately when they interact in the joint activity (i.e., the design in the case of the AI system and the training in the case of the human driver). Of course, in this design of the interaction we cannot forget that the AI system has to be designed to take into account the characteristics of the human being. However, the system also has to be designed to take into account the evidence that the characteristics and functioning of the human cognitive system are not independent of their conditions of interaction (Cañas, 2021). We should also not forget that, from the point of view of ergonomics and human factors, the interest lies in explaining the accountability and, to a certain extent, the responsibility for the results of the interaction between an AI system and a human being. The liability for the results of that interaction is outside the interest of human factors and ergonomics specialists.

In the following sections of this article we will begin by defining interaction as a collaboration between agents. Next, we will briefly point out the ethical implications of this definition of interaction. These ethical implications will require an analysis of the cognitive and non-cognitive components of collaboration between agents, which we introduce in the following sections of the article. Finally, we will point out the implications of this proposal for the design of AI systems.

## Interaction as Collaboration

Traditionally, it has been considered that when we are introducing an automatic machine into an activity, what we are doing is assigning functions that were previously performed by people to a machine that will now be responsible for them. In other words, we are redistributing the functions that people and machines perform in the activity. For this reason, since the beginning of ergonomic studies on automation we have been using the term “function distribution,” so much so that many human factors specialists consider the terms automation and function distribution to be synonymous (Parasuraman et al., 2000).

This traditional way of conceiving the introduction of a machine as a way of assigning functions to the machine in the activity has been useful in many sectors. However, this view is now considered incomplete. The main reason for this incompleteness is that it is basically a way of considering machines only as tools in activities where the subjects are human beings. It should be noted that the term “function sharing” was coined when machines were essentially taking over some of the manual functions for tasks. However, with the assignment of “some intelligence” (cognitive function) to machines, the term “inter-agent collaboration” began to be considered to be more appropriate for referring to a situation in which humans and machines are part of the subject of the activity and share both manual and mental functions. In the activity there may still be machine tools, but some machines with cognitive functions and a certain level of intelligence can no longer be called tools but should now be called “subjects collaborating with human subjects in the performance of the task” (Phillips et al., 2011).

This reasoning leads us to consider that what we should do now is to replace the term “distribution of functions” by the term “collaboration between intelligent machines and people” in order to take into account the fact that people and intelligent machines each take charge of certain functions and share others. In this way we can understand the evidence that already exists that shows that the consequences of an agent's actions depend on the collaboration between agents. Skitka et al. (2000) pointed out that errors of omission might occur when a person fails to respond to a problem that arises in a system because the automatic agent fails to detect the problem or fails to communicate it. There are also errors of commission when a person follows the recommendations given by the automatic agent despite contradictory information being available from other sources. These authors suggested that errors of omission may be due to a decrease in the level of vigilance over what the automated system is doing, and errors of commission might be due to a combination of a failure to heed the advice of the automated system and an erroneous belief in the superiority of the automated system's judgement.

Therefore, if we are to address the ethical issues that result from the introduction of intelligent systems to human activities, we must analyse how the collaboration between intelligent agents should be designed and what the ethics of such a collaboration are. The shift from the point of view of the ethics of two interacting agents, where one is the subject of the activity and one is the tool, to the point of view of the ethics of two agents collaborating in the performance of the activity requires us to rethink how we address the ethical issues of the relationship between human beings and AI.

## Ethics of Collaboration Between Intelligent Agents

Therefore, the question we should ask ourselves now is how the collaboration of people and AI agents should be designed in order for the outcome of such collaboration to be ethically correct. To answer this question, the first thing we must recognize is that, as with any collaboration between agents, the actions of one agent are not independent of the actions of the other agent. The second question is to identify the cognitive and non-cognitive components of this collaboration. In any collaboration between intelligent agents there are two types of component, one that we might call the cognitive type and the other that we might call the non-cognitive type. Although the behavior of an agent, whether human or artificial, is the result of one unique system that processes what is in the environment and responds to the demands imposed on it, it is clear that there are some components of this behavior that could be explained by the way in which information from the environment is processed and others that are related to the motivation or personality of the agents. We call the first components “cognitive” and define them as those components of the system, whether human or artificial, that perceive and process (analyse, elaborate on, memorize, etc.) the information. The latter are called “non-cognitive,” and are those factors that influence the behavior of an agent but do not process information from the environment. These factors should be taken into account because when one agent collaborates with another in an activity, it is important that each perceives and analyses the information and behavior of the other, but it is also necessary that both agents are motivated to collaborate, that the collaboration does not provoke negative emotions for either of them, that there is mutual trust in the collaboration, and so on. Therefore, taking into account this distinction between the cognitive and the non-cognitive components of collaboration between agents, we will start with the cognitive components and then address the non-cognitive components in the subsequent section. However, in addressing them separately, we should not forget that this distinction is only useful as a scientific method for analyzing the agents' behavior. The distinction between cognitive and non-cognitive components does not have a reality independent of the work of the scientist analyzing the agents' behavior. It is evident, for example, that emotions have to be explained by considering how the agent's intelligent system perceives the world.

## Cognitive Components of Collaboration Between Human and AI Agents

It is evident that the interdependence between the agents' actions implies that one agent must understand the actions of the other. If an agent does not understand the actions of another agent with whom he or she is collaborating, he or she may act in a wrong way, causing the outcome of the joint actions to be ethically wrong. The two main factors that affect our understanding of the thoughts and actions of the actors with whom we work are: (1) each actor must pay attention to what the other is doing; and (2) each agent must correctly interpret what the other agent is doing.

We might start by analyzing the communication in the direction of the machine to the person. At all intermediate levels of automation, the person has to know what the machine is doing. To do this, the person must “monitor” the actions of the machine. However, as Stanton (2019) has recently reminded us, people have a great deal of trouble monitoring, and this may be one of the big problems with automation today. In interviews recently conducted by Kyriakidis et al. (2019) with 12 experts in ergonomics and human factors, the interviewees almost unanimously stated that the main problem we have in making automation live up to the expectations it is creating is that we humans are not very good at monitoring processes. For this reason, it is worth spending some time considering the explanations of why monitoring is so difficult for humans. Since monitoring is so essential in the collaboration between people and machines in the design of automation, we must find solutions to ensure that a failure to monitor a smart machine does not lead to a joint action that is ethically wrong.

This poor ability of humans to monitor what a machine is doing has led ergonomics and human factors specialists to investigate the design and use of alarms that warn of adverse events such as incorrect machine actions. Thinking about the collaboration between a person and an AI system, we can ask ourselves what we expect the person to do when hearing the alarm generated by an AI system. As one example, we could expect the alarm to alert the person that something is wrong and that they are required to take control of the activity. For example, in an automatic car when an alarm is heard indicating that the car is drifting off the road, we would expect the alarm to be accompanied by the deactivation of the automatic driving and by the driver taking control of the car to correct the course and avoid the hazard. Conversely, we could have an automatic car in which the alarm serves to warn the driver that something is wrong but the car itself attempts to correct the error, with the driver only being required to monitor what the car is doing. This monitoring could be done by pressing one button, such as a green button, if the driver sees that the car is indeed doing the right thing to correct the error, or another button, such as a red one, if the driver sees that what the car is doing is not the right thing and so the car should try to do something else. Thus, in the first case the alarm means that the driver has to start driving, and in the second case the driver has to monitor what the car is still doing. Mishler and Chen (2018) called the first type of alarm a “direct response” and the second an “indirect response,” and carried out experimental research to compare how long it takes a driver to react to each and how well he or she does so. The results showed that the direct response was faster and more correct than the indirect response. It seems that the indirect response of letting the car remain in automatic mode but requiring the driver to tell the car whether it is doing right or wrong requires more mental resources that take longer and are more prone to error than the direct response of letting the driver start driving immediately by deactivating the automatic system. Thus, in alarms generated by an intelligent system we have a good example of how the actions of the human agent depend on the design of the non-human intelligent agent.

Understanding what the machine is doing is also very important for good collaboration. An example can be found in recent research conducted by Chiou et al. (2021). These authors studied how people interact with robots designed for search and rescue operations. Such robots are currently gaining importance because they have great advantages, including the fact that their use prevents people from having to take risks in search and rescue operations. The people using them can stay in safe places while the robots reach the risk areas.

The use of these robots can be considered as an example of an activity that is carried out in collaboration between a person (the robot operator) and an intelligent device. In this activity it is essential that there is good communication between the robot and the operator; if that communication fails, the intentions of the operator or the robot may be good, but the end result may be wrong. The communication between the robot and the person operating the robot will have a positive effect if they share situational awareness (Endsley and Jones, 2001). By situational awareness we mean “the perception of the elements in the environment within a volume of time and space, the understanding of their significance and the projection of their state into the near future” (Endsley, 1995, p. 36). We can see that when several agents are collaborating in an activity, each must have the situational awareness that is necessary to perform the activities for which he or she is responsible. However, all the collaborating agents need to have shared situational awareness among themselves in order to understand the situation as a whole and the actions that the other agents are taking. This is why we speak of individual situational awareness and shared situational awareness. Without such shared situational awareness, it is possible for one agent, with their own individual, and therefore partial, situational awareness, to carry out an action that is in contradiction with the actions of another agent.

In order to create this shared situational awareness, there needs to be good communication between the actors. Included in this good communication are the explanations that one agent gives to the others about what she/he or it is doing and why she/he or it is doing it. According to Chiou et al. (2021), there are four types of communication between a robot and an operator when we are considering the type of explanation that the robot gives for its actions. First, we have the situation where the robot is designed always to give explanations, without the operator needing to ask for them. Secondly, we could have a robot that only gives explanations when the operator asks for them. In this situation, the operator is left to decide for himself/herself when to ask for explanations, or, in the third type of communication, he/she could be trained to ask for explanations in a convenient way. In the fourth type of communication, which serves as a point of comparison, the robot never gives explanations.

The researchers found that when the robot gives many explanations that are not necessary, the performance of the robot–person team is no better. Unnecessary explanations cause a higher mental load on the operator, which may cause her/him to perform her/his actions badly. Therefore, joint performance is affected by poor robot design. Even if the robot has been designed to act well and explain everything to the operator, such a design results in worse collaboration and thus in a joint performance that may lead to errors. These results clearly indicate that the morality of the individual actions of the agents taken separately does not imply an improvement in the morality of the actions of the robot–human operator system.

Therefore, for the collaboration between the person and the automatic system to occur in an optimal way, it is necessary that the person observes what the automatic system does and understands it. However, in the same way as with collaboration between human agents, good collaboration also requires that the automatic system observes the person's mental state and behavior. The fundamental reason why we need the automatic system to observe the person's state of mind and behavior is that it must be able to adapt to them. Since the mid-1950s, when people began to talk about the distribution of functions, it became clear that this distribution could not be fixed, but instead was dynamic. For this reason, in more recent years, the term “adaptive automatic systems” has come to be used. An adaptive system is defined as a system that can change the type of automation depending on the situation or state of mind and behavior of the person.

The first thing a machine must have is a way of recording the person's actions and interpreting their behavior and, if necessary, their thoughts and mental state. These sensors can be of various types and serve various purposes. First, there are motion sensors. Motion sensors should serve not only to detect the movements of the person interacting with the machine, but also to detect the movements of the people in the environment in which that person and that machine are acting. This would be the case with an automatic car with a driver in charge of certain aspects of driving, when it is driving on a street where there are pedestrians. Vision is the fundamental way in which a person detects the movement of objects and people in her/his environment. For this reason, automated machines are being equipped with image and video recording equipment that is analyzed by software designed to detect movement. Great advances are being made in this field using artificial intelligence methods known as “deep learning” methods (Zhang et al., 2020).

A person's emotional state is very important in interpreting his or her behavior. Emotions are factors that affect a person's decision making and actions. Let us think again about the vehicle situation. All drivers know that our emotional state influences the decisions we make about overtaking, increasing speed, and so on. For this reason, the machine must be able to recognize the emotional state of the person. One option for detecting the emotional state of a person is the use of facial expression analysis software. Munoz-de-Escalona and Cañas (2017) have shown that emotions detected from the analysis of facial expressions can be used to predict how well a task is performed. Another option is pupil diameter. It has been known for many years that pupil diameter is sensitive to the level of arousal caused by the person's emotional state. For this reason, research into how people interact with machines is using systems that measure variations in pupil diameter to analyse a person's emotional processing. The experience of an emotion requires a thought process. For this reason, we consider emotion to be a cognitive factor of collaboration (Lazarus, 1982).

Finally, a topic that is being investigated in the field of intelligent systems design is what has been called “theory of mind” (Carruthers and Smith, 1996; Frith and Frith, 1999). There are two ways of understanding what is meant by a theory of mind. One way is to assume that we all have an “unscientific” idea, based on our experience and our general knowledge of the world, of how the human mind works, in order to predict how the people with whom we interact will behave. Another way to understand what a theory of mind means is to assume that when we interact with other people we have the ability to simulate the minds of those people and thus predict their behavior. This mental simulation of the minds of others is done with the knowledge that we believe we have of the workings of our own minds. It is clear that a theory of mind includes beliefs about ethically correct behavior. The question we must ask in the context of ethics is whether it is possible to design AI systems that have a theory of mind that includes the structure of the human mind and how it functions, and whether people can attribute a mind to AI systems (Winfield, 2018).

## Non-Cognitive Components of Collaboration Between Human and AI Agents

There are many non-cognitive components of agent collaboration, including social cues, security, responsibility, autonomy, and trust (Etemad-Sajadi et al., 2022). We can take as an example the last of these, trust. A fundamental aspect that is currently receiving a great deal of attention in the area of automation is that of human trust in the automated system. In the words of Parasuraman and Riley (1997), trust plays a fundamental role in the disposition of the human being in the automatic systems with which he collaborates in an activity, especially in situations of uncertainty. Researchers such as Lee and See (2004) have shown that when the reliability of the automatic system falls below 90% (i.e., it fails more than 10% of the time), humans stop trusting the automatic system and stop collaborating with it, which affects the effectiveness of the activity they are carrying out.

It is obvious that this effect of human trust in the proper functioning of the automatic system cannot be explained if we think of the automation of an activity as a simple distribution of functions between the human being and the automatic machine. Trust is a factor of social relations. When we talk about trust we are talking about collaboration between social agents. We could say that if we are going to let a Tesla car drive for us, it is because we trust that it will not crash into us or run over a pedestrian. It is not enough to say that we have assigned one or more functions to the car: we need to think that, by letting it drive for us, we are also trusting it to do this well, just as if we were letting someone else drive for us. That person would need to have our trust that he or she would drive well, otherwise we would not let him or her drive for us.

There are different definitions of the term trust that are relevant to our analysis of automation in human activity. Each definition emphasizes different aspects and relates the concept of trust to different types of automated systems and contexts of interaction with them. For example, there is a widely accepted definition proposed by Mayer et al. (1995) that states that trust is the willingness of one party to be vulnerable to the actions of another party, based on the expectation that the other party will perform a particular action, regardless of the ability to control or monitor that other party.

It is clear that there are several important aspects to this definition. The first aspect is the “willingness” of one party to be vulnerable to the actions of the other. Simply put, it can be said that a person voluntarily admits that the actions of another may harm and not always benefit him or her. Therefore, this means that the person who trusts runs a risk of being exposed to negative situations. The second aspect is that the trusting person admits that he/she may not have control over the actions of the person he/she trusts.

Sheridan (2002), one of the most important researchers on the subject of automation, points out that we can consider trust both as an effect and as a cause. As an effect, it refers to how we perceive the reliability of the system. If the system does not fail, if it lets us know what it is doing, if it does what it does with procedures that are familiar to us, if we can predict what it will do in the future, and so on, we will say that the system gives us confidence. As a cause, trust in a system will cause us to behave toward it in a certain way. If we do not trust the system, we will try to avoid it. This is what often happens when we disconnect an alarm that often fails. Because we do not trust it, we switch it off.

This research has found that the reliability of alarms in alerting us to real problems is very important when we talk about trust. It has been shown that if the reliability of an alarm falls below 90%, that is, if it fails more than 10% of the time, people tend to stop paying attention to it. They may even switch it off. If the person notices that every time the alarm goes off there really is a problem with the automation, he or she will tend to pay attention to the alarm every time. On the other hand, when the alarm is triggered by mistake and not because there is something going on to which the person has to pay attention, we have the problem for which we use a phrase taken from the children's story, “the cry wolf effect” (Wickens et al., 2009).

The problem arises after a person experiences the failure of the alarm system several times. The failure of the alarm system is what is called, in the terminology of signal detection theory, a false alarm. The experience of interacting with a machine that has emitted several false alarms leads the person to fail to respond to the alarm when it is correctly triggered. Therefore, the phenomenon that we call the “cry wolf” effect occurs when the person does not respond to a true alarm or responds late because of their experience of many false alarms.

## Improving Collaboration so that It Is Ethically Correct (Acts Ethically)

The question now arises as to how we improve collaboration so that the results of the activity are ethically correct (the results are ethically positive, and ethically negative results are avoided). To answer that question, we have two options. One is for the agents to receive some kind of training to learn how the other agent behaves. In this option, the engineers designing the AI do not need it to behave like a person. It is only necessary for the person to learn how the AI behaves, or the other way round. Another option for engineers is to design the AI to behave as a person would behave. This second option is the one reported by, for example, Kadar et al. (2017). After analyzing some accidents in recent years in which poor interaction between human agents and intelligent automated agents can be identified (the Alvia train crash on the route from Madrid to Santiago de Compostela, the Air France Flight 447 crash and the Asiana Airlines Flight 214 crash), the authors concluded that these accidents occurred largely because the artificial intelligence control strategies for the automated vehicle control were not the same as or similar to those used by human controllers and/or were not used in a similar way. Humans use control strategies based on how we perceive the environment. From the pioneering research of Gibson (1950, 1979), we know that people perceive the environment on the basis of the invariants in it. For example, drivers control their speed through braking, based on the rate of optic expansion (Lee, 1976). However, in AI, kinetic parameters of movements, instead of perceptual invariants, are used to design control strategies. These different control strategies can lead to a lack of understanding between human agents and AI systems, and this lack of understanding can be the cause of accidents.

## Discussion

If the ethics of the actions of collaborative activities involving humans and AI systems are to be explained by the characteristics of that collaboration, it is clear that the methodology for analyzing the ethical issues of those activities must have a basis in the analysis of the characteristics of that collaboration. Even if the actions of each actor are ethically correct in themselves, taken together in the context of the collaboration those actions may lead to ethically incorrect consequences. Therefore, the aim should be to analyse the ethics of collaboration and not the ethics of the individual actions of each agent, whether human or artificial, separately.

We can therefore propose a number of principles to underpin this methodology. First, we must analyse whether the design of the collaboration ensures that there is good monitoring by each agent of the other's actions. If this monitoring is not well-designed, it may happen that the actions of one agent are ignored by the other agent. In that case, even if the actions of each agent are ethically correct in themselves, taken together they might lead to ethically incorrect consequences.

Secondly, We should clarify that our proposal to consider the design of AI systems and the ethical issues in such design must be distinguished from other proposals that are currently receiving much attention. One of these proposals has been referred to by cognitive scientists as “extended cognition.” This idea was popularized by (Clark et al., 1998) and means that automation is explained as an extension of the human mind that provides the human mind with cognitive capabilities that it does not possess without automatic machines. In its most extreme version, this idea would mean treating automatic machines as an extension of the human brain. It would be something like considering that automatic machines are prostheses that are installed in our brains to allow us to perform cognitive activities that we could not do with them. In its less external version, the idea means considering that automatic machines are cognitive artifacts (Hutchins, 1999) or cognitive extenders (Hernández-Orallo and Vold, 2019) that attach themselves to us to perform cognitive activities in such a way that if they disappear we are not able to perform such activities. However, in no case do they become an extension of our nervous system because they are independent entities. It is this independence that makes us consider them as collaborating agents. Automatic machines cannot be extensions of our brain, especially since they are increasingly capable of performing more complex tasks without the intervention of our brains.

This idea of extended cognition has to be considered in the sense that Hutchins (2000) proposed some years ago when talking about how cognitive activities are performed in everyday life. He coined the term “distributed cognition” to refer to the obvious fact that when we perform a cognitive activity such as, for example, memorizing and recalling a fact, we create artifacts that help us to perform it. Consider, for example, how we now remember almost no phone numbers because they are stored in our mobile phone's address book. It is clear that the mobile phone book can be considered as an agent that collaborates with us to memorize and remember phone numbers, but it is not part of our brain. We could give our diary the ability to remember that we have to call a person on a certain day at a certain time, which would give it a certain autonomy from us and allow it to organize our actions, but that does not mean that this diary is part of our brain—it means that we have given a cognitive function to another agent with whom we collaborate. This way of understanding cognitive activities, as proposed by Hutchins, simply means that the activity in which people and machines collaborate is an activity in which the collaboration means that cognitive activity is distributed between human and non-human agents. In no way does it mean that machines become an extension of our brains.

Our proposal is also not what has been called “the human-machine symbiosis approach” to human–machine interaction. In this approach, collaboration between humans and machines is a closed relationship that is mutually beneficial (Licklider, 1960). This symbiosis can take many forms, depending on the type of machine that is collaborating with humans. The collaboration can be dependent if we consider that machines are mere pre-programmed mechanisms that do not possess intelligence. However, in our proposal the collaboration is between agents that can be considered intelligent. Therefore, from the ethical point of view, our proposal goes beyond the symbiosis approach as originally proposed. There may be symbiosis between two agents that are not at the same level of intelligence, but what we are now proposing is a symbiosis in which the collaboration is between two intelligent agents, and that proposal has important ethical consequences.

In conclusion, we should apply the concepts of accountability and responsibility in the context of the collaboration between humans and AI systems, and design AI accordingly. What this means is that the actions of an agent have to be understood as dependent on the collaboration with other agents. In this way, the morality of the system's actions will depend on the morality of an individual agent's actions, but also on the collaboration between the agents. An agent might misinterpret the actions of the other agent and act accordingly, causing the outcome of the actions of the AI–human system to be morally reprehensible. For example, when an intelligent car and its human driver hit a pedestrian we could talk about a morally reprehensible event, but this could be the result of: (1) the driver or the AI system performing a morally reprehensible action (deciding to ignore a traffic light), or (2) the driver misinterpreting the AI system, thinking that it is going to stop the car and doing nothing. There is extensive research about human collaboration that could be applied to this objective.

The idea presented in this article can contribute to achieving the objectives of the ongoing social debate on AI and ethics. For example, understanding the relationship between ethics and AI from the point of view of collaboration between intelligent agents will contribute to the development and application of a strong safety and security practice as defined by Google (2022). This idea can also contribute to the discussion on the ethical principles to be followed in the development of AI according to the ethical guidelines for reliable AI defined by the European Commission (2022). For example, one of these principles is that human beings should be free to make vital decisions for themselves. However, for this principle to be fulfilled, it is necessary that freedom of action be understood in the context of collaboration between intelligent agents.

## Author Contributions

The author confirms being the sole contributor of this work and has approved it for publication.

## Conflict of Interest

The author declares that the research was conducted in the absence of any commercial or financial relationships that could be construed as a potential conflict of interest.

## Publisher's Note

All claims expressed in this article are solely those of the authors and do not necessarily represent those of their affiliated organizations, or those of the publisher, the editors and the reviewers. Any product that may be evaluated in this article, or claim that may be made by its manufacturer, is not guaranteed or endorsed by the publisher.
